# Effect of Tamsulosin on Nocturnal Blood Pressure Load and Dipping Status: A Prospective Ambulatory Blood Pressure Monitoring Study

**DOI:** 10.3390/jcm15187112

**Published:** 2026-09-14

**Authors:** Cumhur Yesildal, Anil Yilmaz, Huseyin Hayit, Nurgul Keskin, Mustafa Kaplan

**Affiliations:** 1 Department of Urology, Sultan 2. Abdülhamid Han Training and Research Hospital, 34668 Istanbul, Türkiye; 2 Department of Internal Medicine, Sultan 2. Abdülhamid Han Training and Research Hospital, 34668 Istanbul, Türkiye; drnurgulkeskin@gmail.com (N.K.);

**Keywords:** BPH, tamsulosin, ambulatory BP monitoring, dipper, non-dipper

## Abstract

**Background:** Alpha-1 adrenergic receptor antagonists are widely used in the management of benign prostatic hyperplasia (BPH) . Their effects on blood pressure (BP) patterns remain incompletely characterized. This study aimed to prospectively evaluate the impact of tamsulosin on ambulatory blood pressure monitoring (ABPM) parameters, with particular focus on nocturnal BP load and dipping status. **Methods:** A total of 83 male patients aged 40–90 years who were initiated on tamsulosin for BPH-related lower urinary tract symptoms (LUTS) were prospectively enrolled; 58 patients with complete, technically adequate paired recordings were included in the final analysis. Participants underwent 24 h ABPM before treatment and at 1 month. Dipping status was defined as a nocturnal BP reduction ≥10%. Pre-post treatment ABPM parameters were compared using paired statistical analyses, and changes in dipping status were evaluated using McNemar’s test. **Results:** Mean 24 h, daytime, and nighttime systolic and diastolic blood pressure (DBP) values did not change significantly after treatment (*p* > 0.05). However, nocturnal systolic and DBP load decreased significantly following tamsulosin initiation (*p* = 0.035 and *p* = 0.005, respectively), along with a reduction in minimum DBP (*p* < 0.001). Changes in dipping status were heterogeneous: 25% of baseline non-dippers converted to a dipper pattern, whereas 36.4% of initial dippers became non-dippers, with no statistically significant overall shift (*p* = 0.81). **Conclusions:** At one month, tamsulosin was not associated with significant changes in mean ambulatory BP. Reductions in nocturnal BP load were observed, but the incremental clinical significance of BP load beyond mean ambulatory BP indices is uncertain. The marked decrease in minimum DBP warrants cautious interpretation as a potential safety signal. These findings should be considered exploratory and require confirmation in controlled studies incorporating earlier and longer follow-up.

## 1. Introduction

BPH is one of the most common causes of LUTS in aging men and represents a major clinical burden due to its progressive nature and impact on quality of life [1]. Alpha-1 adrenergic receptor antagonists are widely used as first-line therapy for BPH-related LUTS, primarily by reducing prostatic smooth muscle tone and improving urinary flow. However, because alpha-1 receptors are also expressed in vascular smooth muscle, these agents may exert systemic hemodynamic effects beyond the lower urinary tract [2].

The magnitude of these vascular effects varies among different alpha-blockers. Non-selective agents such as doxazosin and terazosin are known to reduce systemic BP and may be associated with orthostatic hypotension, whereas uroselective agents such as tamsulosin are generally considered to have minimal impact on BP [3,4]. Nevertheless, this concept of “hemodynamic neutrality” is largely based on office or mean BP measurements and may not fully capture more subtle changes in circadian BP regulation.

ABPM provides a comprehensive assessment of 24 h BP patterns and is considered the gold standard for evaluating circadian BP variability [5]. In particular, nighttime mean BP and the non-dipping pattern have been associated with target-organ damage and adverse cardiovascular outcomes [6]. BP load is a threshold-dependent ABPM measure; its incremental clinical value beyond mean ambulatory BP indices is addressed cautiously in the interpretation of the present findings.

Despite the widespread use of tamsulosin, data regarding its effects on circadian BP parameters remain limited and inconsistent. While some studies suggest minimal hemodynamic influence, others report clinically relevant changes, particularly under specific conditions or patient subgroups. Importantly, most available studies focus on average BP values, with relatively few evaluating nocturnal dynamics and dipping patterns using ABPM.

Therefore, this study aimed to prospectively evaluate the effects of tamsulosin on ambulatory BP parameters, with particular emphasis on nocturnal BP load and dipping status. By using a within-subject ABPM design, we sought to better characterize the potential influence of tamsulosin on circadian BP regulation in patients with BPH-related LUTS.

## 2. Materials and Methods

### 2.1. Study Population and Data Collection

This prospective observational study was conducted between March 2026 and 20 June 2026 after approval by the local Institutional Ethics Committee. Consecutive male patients presenting to the urology outpatient clinic with LUTS secondary to BPH and scheduled to receive tamsulosin treatment were screened for eligibility.

A total of 83 patients aged 40–90 years were initially enrolled. Twenty-five patients were not included in the final paired analysis: 12 did not complete the required follow-up ABPM assessment, 7 had technically inadequate ABPM recordings, and 6 voluntarily withdrew from the study. These exclusions were based on protocol completion or technical adequacy and were not based on observed BP values or treatment response. A complete, analysis-ready set of major baseline characteristics was not available for all 25 excluded participants because some baseline assessments were incomplete and some individuals declined to continue study participation. Consequently, a formal included-versus-excluded baseline comparison could not be performed. Fifty-eight patients were included in the final analysis. Written informed consent was obtained from all participants prior to enrollment.

Eligible patients were required to have a diagnosis of BPH-related LUTS and be scheduled to initiate tamsulosin monotherapy. Exclusion criteria included a history of prostate or urethral surgery, suspected or confirmed prostate cancer, neurogenic bladder, neurological disorders affecting lower urinary tract function, circadian rhythm disorders, active urinary tract infection, newly diagnosed hypertension, severe chronic renal or hepatic insufficiency, severe cardiac arrhythmias potentially interfering with ABPM recordings, and any change in the class or dosage of antihypertensive medications within the preceding three months.

### 2.2. Ambulatory Blood Pressure Monitoring

All participants underwent 24 h ABPM before initiation of tamsulosin therapy and again after one month of treatment. BP measurements were obtained using a validated oscillometric ambulatory blood pressure monitor (ABPM-50/PM-50; Contec Medical Systems Co., Ltd., Qinhuangdao, China).

The device was programmed to record measurements every 20 min during daytime hours (07:00–23:00) and every 30 min during nighttime hours (23:00–07:00) [7], in accordance with current recommendations for ABPM. Participants were instructed to maintain their usual daily activities while avoiding excessive physical exertion, alcohol consumption, and excessive caffeine intake during the monitoring period.

All ABPM recordings were reviewed and interpreted by an internal medicine specialist who was not involved in patient management.

BP load was defined as the percentage of BP measurements exceeding accepted ambulatory BP thresholds during the monitoring period. Daytime systolic and diastolic BP load were calculated using thresholds of 135 mmHg and 85 mmHg, respectively, whereas nighttime systolic and diastolic BP load were calculated using thresholds of 120 mmHg and 70 mmHg, respectively.

The nocturnal BP dipping rate was calculated using the following formula:

$$\frac{\mathrm{Mean}\;\mathrm{Daytime}\;\mathrm{BP}-\mathrm{Mean}\;\mathrm{Nighttime}\;\mathrm{BP}}{\mathrm{Mean}\;\mathrm{Daytime}\;\mathrm{BP}}\times 100$$

Patients demonstrating a nocturnal BP reduction of ≥10% were classified as dippers, whereas those with a reduction of <10% were classified as non-dippers [8].

### 2.3. Treatment Protocol and Follow-Up

Baseline ABPM was completed before tamsulosin initiation. All patients then received tamsulosin 0.4 mg once daily as monotherapy for BPH-related LUTS and were followed prospectively for one month. At the end of the treatment period, a second 24 h ABPM assessment was performed using the same monitoring schedule and predefined daytime and nighttime thresholds as at baseline. Patients who did not complete the required follow-up ABPM, had technically inadequate recordings, or voluntarily withdrew were not included in the paired analysis. Participants with established cardiovascular disease, diabetes mellitus, or chronic kidney disease continued their routine clinically indicated follow-up and treatment; disease-specific therapy was not modified for the purposes of this study.

To minimize potential confounding effects on BP measurements, no changes were made to the class or dosage of antihypertensive medications during the study period. Patients receiving antihypertensive therapy at baseline continued the same treatment regimen throughout follow-up, whereas no participant was initiated on a new antihypertensive medication between baseline and post-treatment ABPM assessments.

Because of the prospective before-and-after study design, each participant served as his own control.

### 2.4. Study Outcomes

The primary outcome of this study was the change in nocturnal BP load following tamsulosin treatment.

Secondary outcomes included:

- Changes in 24-h, daytime, and nighttime mean BP values;
- Changes in dipping status;
- Variations in BP variability parameters;
- Changes in ambulatory arterial stiffness index.

### 2.5. Statistical Analysis

Statistical analyses were performed using SPSS software version 24. Continuous variables were expressed as mean ± standard deviation or median (minimum–maximum), as appropriate. Categorical variables were presented as frequencies and percentages.

Normality of distribution was assessed using the Shapiro–Wilk test. Pre- and post-treatment continuous variables were compared using the paired samples *t*-test. Changes in categorical variables, including dipper status, were evaluated using McNemar’s test.

For subgroup analyses based on changes in dipping status (dipper–dipper, dipper–non-dipper, non-dipper–dipper, non-dipper–non-dipper), one-way analysis of variance (ANOVA) was used. Additionally, analysis of covariance (ANCOVA) was performed to adjust for baseline values where appropriate.

All statistical tests were two-tailed, and a *p*-value < 0.05 was considered statistically significant. Subgroup analyses were considered exploratory.

## 3. Results

### 3.1. Baseline Characteristics

Of the 83 enrolled patients, 25 were not included in the final paired analysis: 12 did not complete the required follow-up ABPM assessment, 7 had technically inadequate ABPM recordings, and 6 voluntarily withdrew. These exclusions were procedural and were not based on observed BP values or treatment response. Because a complete set of major baseline variables was not available across all excluded participants owing to incomplete baseline assessments and withdrawal from further study participation, a valid formal comparison with the analyzed cohort could not be performed. The remaining 58 patients constituted the final analysis population. Their mean age was 61.6 ± 7.99 years, with a median of 60.0 years (range: 43–86). (Table 1).

### 3.2. Changes in Ambulatory Blood Pressure Parameters

No significant differences were observed between pre- and post-treatment measurements in mean 24 h, daytime, or nighttime systolic and DBP values (*p* > 0.05 for all comparisons).

In contrast, significant reductions were detected in parameters reflecting nocturnal BP burden. Nighttime systolic BP load decreased from 64.7 ± 31.2% to 53.8 ± 32.5% (*p* = 0.035), and nighttime DBP load decreased from 51.2 ± 34.5% to 38.4 ± 33.1% (*p* = 0.005). Additionally, minimum DBP values were significantly lower after treatment (52.4 ± 10.8 mmHg vs. 42.5 ± 11.8 mmHg, *p* < 0.001).

No statistically significant changes were observed in daytime BP load, BP variability indices, or AASI (*p* > 0.05 for all) (Table 2).

### 3.3. Changes in Dipping Status

At baseline, 37.9% of patients were classified as dippers and 62.1% as non-dippers. After one month of treatment, 39.7% were classified as dippers and 60.3% as non-dippers.

Among patients initially classified as non-dippers, 25.0% converted to a dipper pattern, whereas 36.4% of initial dippers transitioned to a non-dipper pattern. Overall, these changes were heterogeneous and not statistically significant (*p* = 0.81) (Table 3).

### 3.4. Subgroup Analyses Based on Dipping Status

Patients were categorized into four groups based on changes in dipping status: persistent dippers, persistent non-dippers, dipper-to-non-dipper, and non-dipper-to-dipper.

Post-treatment nighttime systolic and DBP values differed significantly between these groups (*p* = 0.003 and *p* = 0.005, respectively) (Table 4).

### 3.5. ANCOVA Analysis

After adjustment for baseline values using ANCOVA (Analysis of Covariance), no significant group effect was observed for 24 h or daytime mean BP parameters (*p* > 0.05).

However, a significant group effect persisted for nighttime systolic and DBP (*p* = 0.006 and *p* = 0.004, respectively), as well as for systolic and diastolic nocturnal dipping rates (*p* < 0.001) (Table 5).

## 4. Discussion

BPH and hypertension are two common conditions in the aging male population, with sympathetic overactivity contributing to the pathophysiology of both disorders [9,10]. In this prospective within-subject study, tamsulosin treatment was not associated with significant changes in 24 h, daytime, or nighttime mean ambulatory BP. Reductions in nocturnal BP load and a marked decrease in minimum DBP were observed. Because BP load has uncertain incremental clinical value beyond mean ambulatory BP, these findings should be interpreted as exploratory rather than as evidence of a selective cardiovascular benefit. The decrease in minimum DBP also merits attention as a potential safety signal.

Non-selective alpha-blockers such as doxazosin and terazosin are known to produce clinically meaningful reductions in systemic BP. In a large-scale study involving 1175 patients, these agents were associated with an average reduction of approximately −8/−5 mmHg [11], which may increase the risk of orthostatic hypotension and syncope, particularly in normotensive individuals with BPH. In contrast, the absence of a significant change in mean ambulatory BP parameters in our study (*p* > 0.05) supports the uroselective profile of tamsulosin. This observation is consistent with the findings of de Mey et al., who demonstrated that tamsulosin did not significantly alter ABPM measurements, whereas terazosin was associated with a pronounced hypotensive effect [12].

ABPM is considered the gold standard for assessing cardiovascular risk, masked hypertension, and circadian BP variability, including dipping patterns [13,14]. Previous studies have shown that non-selective alpha-blockers, such as doxazosin, may influence circadian BP profiles by converting non-dipper patients to a dipper pattern and attenuating morning BP surges [15,16]. However, data regarding the effects of uroselective agents such as tamsulosin on circadian BP dynamics remain limited and inconsistent. While some reports suggest minimal hemodynamic impact, others describe clinically significant hypotensive events requiring medical attention [17,18].

Evidence from other functionally uroselective alpha-1 blockers further illustrates that hemodynamic responses depend on the study population. Mondaini et al. reported that alfuzosin 10 mg did not meaningfully affect BP in young, healthy, normotensive men [19]. In a multicenter prospective cohort, Zhang et al. found that alfuzosin 10 mg was generally well tolerated in men with BPH/LUTS with or without concomitant antihypertensive therapy, although greater BP reductions were observed in patients with untreated or uncontrolled hypertension [20]. Because these studies evaluated alfuzosin, they provide relevant class context but should not be interpreted as direct evidence for tamsulosin.

The statistically significant reductions in nocturnal BP load should not be interpreted as an established reduction in cardiovascular risk. In an outcome-based analysis of 8711 participants, Li et al. found that BP load did not improve cardiovascular risk stratification beyond mean 24 h BP levels [21]. A subsequent review likewise found no clear or consistent incremental value of BP load over mean ambulatory BP indices in adults [22]. Because BP load is threshold-dependent, it may change without a parallel change in mean BP. Accordingly, the present BP-load findings are hypothesis-generating, and their clinical significance remains uncertain.

Nocturia and sleep disruption are also relevant potential determinants of nighttime BP. Population-based data have demonstrated an association between nocturia and hypertension [23], and recent sleep-BP data suggest that nocturia may influence BP during sleep independently of readings obtained during nocturnal activity [24,25]. Although improvement in nocturia may occur after alpha-blocker therapy, the magnitude of symptomatic response is heterogeneous and cannot be assumed for every participant. Nocturia frequency and sleep quality were not prospectively recorded before and after treatment in the present study; therefore, their potential mediating or confounding effects could not be analyzed. The absence of a significant change in mean nighttime BP consequently cannot be interpreted in relation to an assumed improvement in nocturia.

The timing of ABPM also limits safety interpretation. Large observational data indicate that the risk of severe hypotension is greatest during the first weeks after initiating or restarting tamsulosin [26]. Because ABPM was repeated only at one month, transient early hypotensive episodes may have been missed. The reduction in minimum DBP from 52.4 to 42.5 mmHg therefore warrants cautious attention, even though mean 24 h, daytime, and nighttime BP did not change significantly. Minimum BP is sensitive to transient or isolated readings, and the absence of early repeated ABPM and symptom-linked measurements prevents assessment of the duration and clinical consequences of these nadirs.

The eligibility range of 40–90 years was intentionally broad to reflect the clinically relevant spectrum of middle-aged and older men treated with tamsulosin for BPH-related LUTS; the observed age range was 43–86 years. However, age-related differences in arterial stiffness, autonomic reserve, comorbidity burden, and concomitant medication use could modify hemodynamic response. Because the sample size did not support reliable age-stratified inference, the influence of the youngest and oldest participants cannot be excluded, and the findings should not be assumed to apply uniformly across the entire eligibility range.

Established cardiovascular disease, diabetes mellitus, and chronic kidney disease are clinically relevant potential modifiers of BP response. Participants with these diagnoses continued routine clinically indicated follow-up and treatment, and no disease-specific therapy was altered for study purposes. However, the present study was designed for paired analysis of the overall cohort and was neither prespecified nor powered for reliable comorbidity-stratified inference. Given the total sample size, overlap among these conditions and small subgroup cells would be expected to produce unstable post hoc estimates. We therefore did not present potentially misleading subgroup effects. A future secondary analysis with a prespecified statistical plan is intended when a sufficiently complete comorbidity and treatment dataset is available for an analysis that accounts for overlapping conditions.

In exploratory ANCOVA, differences among the four dipping-transition groups persisted for nighttime systolic and DBP and for nocturnal dipping rates after adjustment for baseline values. However, these groups were defined by observed changes in dipping status and contained relatively small numbers of participants. The findings should therefore not be interpreted as evidence that tamsulosin produced a specific group-dependent hemodynamic effect.

Transitions between dipper and non-dipper patterns were heterogeneous and not statistically significant. This variability is consistent with the limited short-term reproducibility of categorical dipping classification. Neither the categorical transitions nor the accompanying exploratory subgroup findings establish a treatment-related improvement in circadian BP regulation.

Insufficient nocturnal BP reduction has been associated with target-organ damage and cardiovascular risk; however, that evidence should not be extrapolated to infer that a reduction in BP load alone is beneficial. In the absence of a parallel reduction in mean nighttime BP, a control group, or clinical cardiovascular outcomes, the observed BP-load changes cannot establish favorable modulation of nighttime hemodynamic stress.

## 5. Key Findings and Limitations

In this prospective within-subject study of 58 men receiving tamsulosin 0.4 mg daily for one month, mean 24 h, daytime, and nighttime ambulatory BP remained unchanged. Nocturnal systolic BP load decreased from 64.7% to 53.8% (absolute change −10.9 percentage points; *p* = 0.035), and nocturnal DBP load decreased from 51.2% to 38.4% (absolute change −12.8 percentage points; *p* = 0.005). Minimum DBP declined from 52.4 to 42.5 mmHg (*p* < 0.001), whereas dipping status did not change significantly (37.9% vs. 39.7% dippers; *p* = 0.81). The BP-load findings are statistically significant but clinically uncertain, while the minimum-DBP reduction warrants consideration as a potential safety signal rather than being incorporated into a claim of overall hemodynamic neutrality.

This study has several limitations. First, it was conducted at a single center with a relatively small sample size (*n* = 58), which limits generalizability and precluded reliable age-stratified inference across the broad 40–90-year eligibility range. Second, there was no placebo or active comparator group; consequently, regression to the mean, day-to-day ABPM variability, and other time-related influences cannot be separated from treatment-associated changes. Third, 25 of 83 enrolled patients did not contribute paired outcomes because they did not complete follow-up ABPM, had technically inadequate recordings, or voluntarily withdrew. These exclusions were procedural and unrelated to observed treatment response, so no outcome-based selective exclusion occurred. Nevertheless, complete major baseline data were unavailable across the excluded cohort because of incomplete assessments and withdrawal from further participation; therefore, comparability could not be empirically verified and residual attrition-related bias cannot be entirely excluded. Fourth, this study was neither prespecified nor powered for analyses stratified by established cardiovascular disease, diabetes mellitus, or chronic kidney disease. The overlap among these conditions and the anticipated small subgroup cells would have made post hoc estimates unstable; a future secondary analysis with a prespecified statistical plan is intended when a sufficiently complete comorbidity and treatment dataset is available. Fifth, ABPM was repeated only at one month, potentially missing transient hypotension during the early treatment period. Sixth, nocturia frequency and sleep quality were not recorded, preventing evaluation of their mediating or confounding effects on nighttime BP. Seventh, minimum DBP may be influenced by isolated readings, and symptom-linked or orthostatic measurements were unavailable to clarify its clinical relevance. Finally, multiple ABPM-derived parameters were evaluated; dipping status limited short-term reproducibility, and the dipping-transition subgroup findings should be regarded as exploratory. Controlled studies incorporating early ABPM, symptom and sleep assessment, comorbidity-specific evaluation, and longer follow-up are needed.

## 6. Conclusions

After one month of tamsulosin treatment, mean 24 h, daytime, and nighttime ambulatory BP did not change significantly. Although nocturnal systolic and diastolic BP load decreased, current evidence does not support interpreting BP load reduction alone as an established cardiovascular benefit or as proof of a selective improvement in circadian hemodynamics.

The marked decrease in minimum DBP warrants cautious clinical interpretation as a potential safety signal. Because this study did not include early repeat ABPM, symptom-linked measurements, nocturia or sleep assessment, or a control group, transient early hypotension and non-treatment-related variability cannot be excluded.

In clinical practice, the unchanged mean ABPM values are reassuring at the group level, but clinicians should remain attentive to dizziness, orthostatic symptoms, or hypotension during treatment initiation, particularly in older patients and those with cardiovascular, renal, or metabolic comorbidities or concomitant antihypertensive therapy. Larger controlled studies with early and longer-term monitoring are required to determine the clinical meaning of the observed BP-load and minimum-DBP changes.

## Figures and Tables

**Table 1:** Baseline characteristics.

Variable	Value (*n* = 58)
Age (years), mean ± SD	61.6 ± 7.99
Age (years), median (min–max)	60.0 (43–86)

**Table 2:** Comparison of ABPM parameters before and after treatment.

Parameter	Pre-Treatment	Post-Treatment	*p*-Value
24-h Mean SBP (mmHg)	124.8 ± 11.2	126.1 ± 10.5	0.305
24-h Mean DBP (mmHg)	78.4 ± 9.1	79.2 ± 8.4	0.437
Daytime Mean SBP (mmHg)	126.5 ± 11.4	127.8 ± 10.8	0.391
Daytime Mean DBP (mmHg)	80.2 ± 9.5	80.7 ± 8.9	0.674
Nighttime Mean SBP (mmHg)	121.2 ± 14.8	121.2 ± 13.9	0.999
Nighttime Mean DBP (mmHg)	73.4 ± 10.2	75.2 ± 10.6	0.237
Day SYS Load (%)	24.8 ± 20.3	26.4 ± 21.1	0.487
Day DIA Load (%)	26.1 ± 22.4	24.5 ± 20.8	0.367
Night SYS Load (%)	64.7 ± 31.2	53.8 ± 32.5	0.035 *
Night DIA Load (%)	51.2 ± 34.5	38.4 ± 33.1	0.005 *
Minimum DBP (mmHg)	52.4 ± 10.8	42.5 ± 11.8	<0.001 *

* Statistically significant.

**Table 3:** Changes in dipping status.

	Post-Treatment Dipper	Post-Treatment Non-Dipper	Total
Pre-treatment Dipper	14 (24.1%)	8 (13.8%)	22 (37.9%)
Pre-treatment Non-dipper	9 (15.5%)	27 (46.6%)	36 (62.1%)
Total	23 (39.7%)	35 (60.3%)	58 (100%)

*p* = 0.81 (McNemar test).

**Table 4:** Comparison of nocturnal BP between groups (ANOVA).

Parameter	Pre-Treatment *p*	Post-Treatment *p*
Nighttime Mean SBP	0.339	0.003 *
Nighttime Mean DBP	0.522	0.005 *

* Statistically significant.

**Table 5:** ANCOVA results.

Parameter	Group Effect (*p*)
24-h Mean SBP	0.374
24-h Mean DBP	0.086
Nighttime Mean SBP	0.006 *
Nighttime Mean DBP	0.004 *
SYS Night Dipping	<0.001 *
DIA Night Dipping	0.004 *

* Statistically significant.

## Data Availability

Data available on request due to privacy/ethical restrictions.
